# Perceived Competence in the Face of Death before and after Nursing Studies: An Intrasubject Longitudinal Study

**DOI:** 10.3390/ijerph182212084

**Published:** 2021-11-17

**Authors:** Enrique Sáez-Alvarez, Pilar Medrano-Abalos, Cristina Cunha-Pérez, Jesús Cuesta-Fernández, Salvador Martín-Utrilla

**Affiliations:** 1 Nursing Department, Catholic University of Valencia “San Vicente Mártir”, 46007 Valencia, Spain; pilar.medrano@ucv.es (P.M.-A.); cristina.cunha@ucv.es (C.C.-P.); salvador.martin@ucv.es (S.M.-U.); 2 Research Group in Palliative Care, Catholic University of Valencia “San Vicente Mártir”, 46007 Valencia, Spain; cuesta_jesfer@gva.es

**Keywords:** palliative care, communication skills, self-care, compassion, nursing students, death

## Abstract

Perceived competence is a subject’s perception of being able to interact effectively with the environment. Perceived Competence in the face of death in Nursing degree programs in which the presence of the subject of death and bereavement is key becomes more topical and relevant. The objective of this study is to determine whether this competence is improved through Nursing Studies. This study was designed as paired repeated intrasubject measures, initial measurement at the beginning of the first year and second measurement in the fourth year of the Nursing degree. One hundred and seventeen nursing students were assessed. Significant improvement is evidenced in three of the four dimensions of Perceived Competence in the face of death (Accompaniment and Communication: 25.70/29.34; Self-Confidence: 9.64/12.78; Management of Self-Fear: 13.18/14.66). These results show notable differences with their cross-sectional predecessors, suggesting the need for further studies in this field to consolidate a still developing body of knowledge.

## 1. Introduction

The Perceived Competence construct, Nogas, Schweitzer and Grumet [1] refer to it as “sense of competence”, can be defined as the perception that one is able to interact with the environment effectively [2]. The study of competence in the face of death is necessary for the choice of the areas in which to interact [3] and for the effective performance of the tasks and processes of caring for people at the end of life. There seems to be a consensus among some authors [4,5,6] about recognizing the strong relationship between perceived control and emotional well-being. It is believed that, regardless of whether control is exercised, the belief in control makes people feel capable of relating effectively to their environment. This control belief influences in adaptation to the environment through two complementary mechanisms: one of them promotes direct action to alter situations that are negative for the subject and the other facilitates the emotional response. Both mechanisms make these actions more effective from the perspective of the individual [7].

Even though the study of Perceived Competence in the face of death is becoming more topical and relevant [8,9,10,11,12] as well as the fact that awareness is growing regarding the urgency of its study in Nursing degree programs in which the presence of the subject of death and bereavement is key, the number of research studies and, therefore, their methodological variety is still not sufficiently wide to provide robustness of the results. The nursing student must be prepared to face situations related to palliative care and the end of life to respond adequately to these circumstances. In addition, professionals must know how to handle information related to bad news to the family and the patient throughout the end-of-life process. Therefore, much of the literature reviewed explores the construct of Perceived Death Competence through cross-sectional designs in which comparisons are made between students of nursing or other health specialties of different academic levels [8,9,13,14,15,16,17], postgraduate nursing staff [10], students of various specialties among themselves [10,12,17,18], or from different universities [13,16]. In addition, when reviewing the rest of the scientific production, it was observed that the cross-sectional studies conducted evaluated the change in the level of Perceived Death Competence through short specific training programs lasting between four and forty-five hours [13,16,17,19,20,21,22].

However, when it comes to the question of whether training programs (both specific and long-term) increase Perceived Death Competence, the answer is not easy. In the study conducted by Colell [13] with more than 400 subjects, although comparisons are made cross-sectionally between the first, second, and third years of nursing courses at two Spanish universities, no relevant results are found for most of the variables used to analyze competence (either with the EBAM scale, the Wallston Perceived Competence scale, or the Self-Reliance in the Face of Death scale). Similarly, Schmidt [16] in a study with first, second-, and third-year subjects (from two countries, three universities, and five different degrees) found differences in the Bugen scale scores with statistical significance between them, with lower scores as the degree course progressed; unfortunately, the author did not carry out this analysis according to the studied specialty. Sáez et al. [15], on the other hand, comparing fourth and sixth year medical students, found no differences between the two groups, nor between first and second year nursing students [23] using the Bugen Brief Modified Death Competence Scale [24]; they also found no differences between first and third year nursing students using the Bugen scale, although there were differences in the Post-Mortem Care dimension of the Medrano Perceived Competence scale [25], with higher scores in first year students.

A significant shift from the above-mentioned question is made when it comes to evaluating short training programs. Within this second category, Schmidt et al. [17] using a sample of 87 health science students after a 45-h course, Claxton-Oldfield, Crain, and Claxton-Oldfield [21] with 17 palliative care volunteers in a 27-h training program, Colell [13] comparing a sample of 176 first-year nursing students before and after a specific four-hour course on dying and end-of-life care, or Brysiewicz and Mcierney, in South Africa [19], studying a group of health care workers before and after a three-day course consistently reported higher scores on Perceived Competence in the face of death after the training period.

Given the disparity of results found in the literature, a question arises as to whether an intrasubject methodology, from the time students begin their Nursing degree until the end of their studies, could shed light on students’ perception of competence, offering a clearer perspective and a closer approximation to the reality of the efficiency of regulated studies.

## 2. Materials and Methods

The study is part of a larger longitudinal study that aimed to provide an overview of attitudes to death in new students entering the Nursing degree, which began in 2016. Participants’ responses were coded so that after three years the same questionnaires were administered again to those students and their responses at both time points could be matched. Therefore, it is a paired repeated measures intrasubject design, with an initial measurement at the beginning of the first year of studies (September) and the second measurement in February of the fourth year of the Nursing degree.

A convenience sample was used, consisting of all first-year students of the Faculty of Nursing at the Catholic University of Valencia, who were administered the EBBCAM (Escala Breve de Bugen de Competencia ante la Muerte) scale of Perceived Competence in the face of death at the beginning of the academic year. Those same subjects were administered the same scale halfway through their fourth year of studies.

The EBBCAM Scale consists of 16 items with a range of scores between 10 and 70, the highest scores indicating greater Perceived Competence in the face of death, and four dimensions: Accompaniment and Communication, Post-Mortem Care, Self-Confidence, and Self-Fear Management, all of which assess areas of interest within the field of Death Competence. This scale has shown adequate psychometric validity (Chi2: 256.3268/0.000; df: 98; NFI: 0.920; NNFI: 0.937; CFI: 0.949; IFI: 0.949; RMSEA: 0.05) and reliability (Cronbach’s Alpha 0.84) [20].

For the statistical treatment of the data, the reliability study was carried out, first by analyzing internal consistency through Cronbach’s alpha. Subsequently, the normality of the data was determined using the Kolmogorov–Smirnof statistic, from which it was decided to adopt Pearson’s r test for correlational analysis and the student’s t test for repeated measures for comparative analysis between participants when they were in 1st and 4th grade. For comparisons by sex, Student’s t was used for independent samples. Finally, to estimate the scores of the students in the fourth year, based on those obtained in the first year, a simple linear regression analysis was carried out.

A minimum required significance level of 0.05 was considered in all analyses. Data analysis was carried out using the statistical program SPSS v23 (IBM Corp., Armonk, NY, USA).

To verify that the influence of experimental loss is minimal or null, the total group analyzed in the first year (*n* = 251) was compared with the final sample (*n* = 117) in the areas of Accompaniment and Communication, Post-Mortem Care, Self-Confidence, and Self-Fear Management, using paired samples t test, with no evidence of differences between the two groups in any of the variables analyzed. Likewise, a Student’s t for independent samples was carried out for high and low scores in competition, (greater than 40 or less than 30 points respectively) by sex and age in the four dimensions without finding differences with statistical significance between both groups.

**Figure 1:**
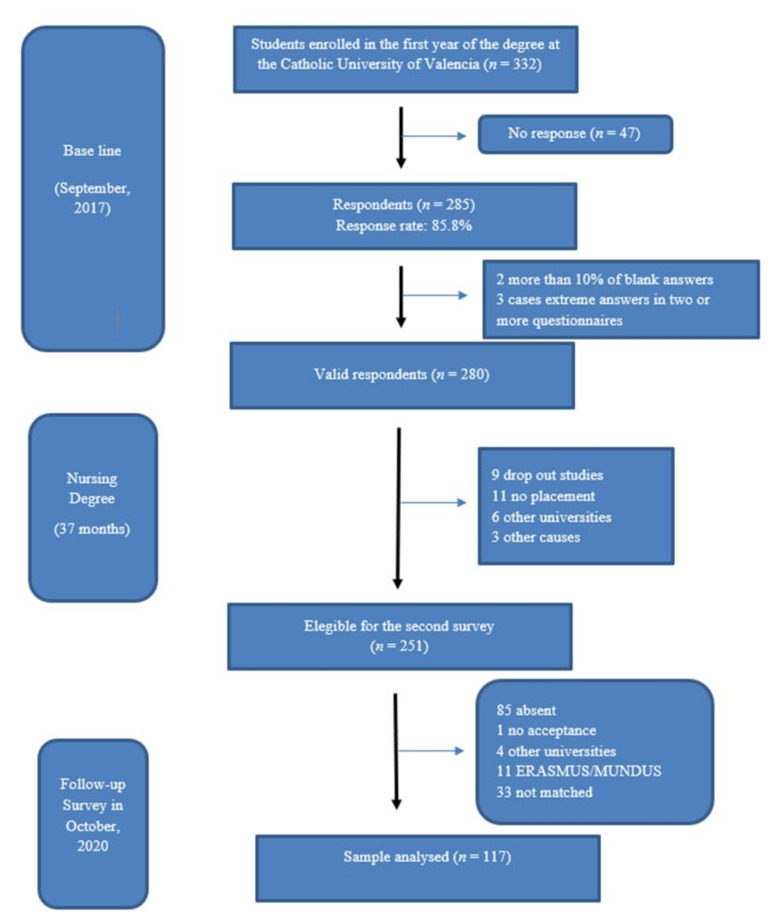
Participant flow of the present study. ERASMUS/MUNDUS: EuRopean/World Community Action Scheme for the Mobility of University Students.

Out of 251 students (Figure 1) who answered the questionnaire in the first year of their degree, 117 students also answered the questionnaire in the fourth year, which represents a sample loss of 168 participants (46.61%).

## 3. Results

To determine the consistency of the scale, Cronbach’s alpha coefficient was calculated. The EBBCAM scale shows high internal consistency measures on all dimensions in both first and fourth years of the degree, except for Self-Fear Management for first year students where internal consistency is moderate (Table 1). 

Table 2 shows a description of the sample. Despite the loss of 168 between first- and fourth-degree students, a total number of 117 participants were included. The ratio of female to male nursing students in our school is approximately 80:20. This ratio is to be considered when explaining the differences in the sample obtained, with a higher participation of the female group. We also found high interest in death in general and disparate levels of Perceived Death Competence in both the first and third years of the degree.

The next step was to consider the differences between female and male participants in the different dimensions of the EBBCAM scale. No gender differences were found in Perceived Competence in the face of death in any of these dimensions (Table 3).

When we consider changes in students’ perceived competence between the first- and fourth- year of Nursing degree, significant differences were found in three of the four dimensions of the EBBCAM scale (Table 4). Higher scores are observed in fourth year students compared to first year students in the dimensions of Accompaniment and Communication, Self-Confidence, and Self-Fear Management.

A simple linear regression model was calculated to explain the relationship between the scores in year 1 and 4 (Table 5). Four independent variables were tested (Age, Interest, Gender, and global Perceived Competence), but only Perceived Competence in the first year is consistent with the regression model.

Table 6 shows that 13.2% of the score in fourth year could be explained by the regression model, and there is statistical evidence that the regression model fits the data. Likewise, there is statistical evidence that Perceived Death Competence in first year students influences scores in Perceived Death Competence among students in fourth grade. On the other hand, the predictive model based on the four components of Perceived Competence (Accompaniment and Communication, Post-Mortem Care; Self-Confidence and Self-Fear Management) shows relevance in all dimensions, except Self-Fear Management (*p* > 0.05).

In both the first- and fourth-degree course, no correlation was found between the age of the participants and the different dimensions of Perceived Competence in the face of death (Table 7). Interest is statistically significantly related, mainly in the first degree-course.

## 4. Discussion

This study’s main contribution is the finding that the programmed training of students of the Nursing degree improves their Perceived Competence. As these students declare that the care of the dying patient and his death, as well as dealing with family members, are one of the most difficult situations they must face, the results obtained are clearly relevant. Previous research reported either no difference between the first- and last-degree courses or even higher scores on Perceived Death Competence for the students of the first year of degree. This has been the case in the scientific literature to such an extent that some researchers [16,26] have argued that this decrease in scores as the experience and contact with the person who is dying progresses, and along with the reality of the event and “facing it”, was a consequence of the confrontation with reality, which is more threatening than the previous expectation that the subjects might have had; and, on the basis of the available data, such an explanation seemed so plausible that the term “Illusion of Competence” was coined to refer precisely to this fact [18,24]. However, this line of research has shown that the aforementioned argument no longer could be solidly sustained since the results presented in the present study point precisely in the opposite direction, demonstrating that the more experience the student has acquired, the greater the sense of competence the subject shows. Although future confirmatory studies should be carried out to determine what causes the stabilization or decrease in Perceived Competence scores in the between-subjects’ studies, the design presented in the present research has some advantages, for example, the pre- and post-intervention matching which eliminates biases such as cultural bias, family influence, or age, as shown in the results, and does not correlate with the results. Additionally, unlike what might occur with short term pre- and post-intervention studies, whose results could be attributed to phenomena such as experimenter bias or social desirability bias, both could be diluted by the duration of the program and its integration into the lives of the students, as it consists not only of theoretical content but also of practical, non-punctual, continuous content in which change and integration would occur in such a way that the participant could not attribute it to any specific action.

Regarding the results, differences can be seen in three of the four dimensions of Perceived Competence in the face of death, with the Postmortem Care dimension being the only one in which participants report no improvement. This finding is meaningful considering that this may be the area in which the students are least involved during their practical studies, since, as health care is designed in Spain, such care is not carried out entirely at the deceased’s place of death, limiting the student’s training opportunities. It would be desirable to carry out further studies of these results in cultural environments where such care is promoted at a more educational level among the nursing community. Besides, the predictive model indicates that Perceived Competence in the first year of the degree can predict part of the Perceived Competence at the end of the studies in a low percentage which, although statistically significant, confirms the existence of other unmeasured variables such as, for example, the students’ own academic curriculum and their practical experience, so that designing a study which would consider this fact would be the next logical step.

Other interesting results are, firstly, the fact that there is no influence between gender and Perceived Competence in the face of death, which has been previously reported [16,18,27,28] or that interest in death is only related to Perceived Competence when the students are in the initial phase of their degree, and only regarding the dimension of Self-Confidence when they are in the final stages of their studies.

The relevance of this study lies on the one hand, in the need to implement longitudinal studies involving repeated measurements on the same subjects and, on an academic level, in the discovery of the fact that post-mortem care is an area for improvement, with the implications and didactic changes that could help to achieve that goal. On the other hand, the fact that students have perceived an increase in all other areas, and that this increase is probably not due to the effect of maturation by age, indicates that the training plans have a positive effect, encouraging the continuation of this line of education.

The main limitations of this study, however, consisted, on the one hand, in the fact that it was necessary to wait three years to analyze the same subjects again and to avoid suffering an excessive experimental loss, which in this case was more than fifty percent of the participants, and, on the other hand, regarding the measuring instruments. Although the Brief Scale of Perceived Competence in the Face of Death improves its predecessors at a psychometric level [20] and is a suitable tool for rapid screening, it is not sufficiently broad to cover the complexity of the areas it measures nor the totality of those that comprise the spectrum of possible areas of nursing staff competence, including, of course, students. Finally, it is important to bear in mind that since the study plans of each university may differ in certain aspects, care should be taken in generalizing these results and new research work will be necessary to exhaustively guarantee such relationship.

## 5. Conclusions

Programmed training of students of the Nursing degree improves their Perceived Competence in the face of death. The results obtained from an intrasubject measurement over the years in Nursing Studies reflect the changes in this area. Differences are found in three of the four dimensions of Perceived Competence in the face of death: Accompaniment and Communication, Self-Confidence, and Self-Fear Management. In our study there is no influence between gender and Perceived Competence in the face of death.

## Figures and Tables

**Table 1:** Internal consistency analysis of the EBBCAM scale.

	Cronbach’s Alpha 1st Year	Cronbach’s Alpha 4th Year
Accompaniment and Communication	0.78	0.83
Post-Mortem Care	0.70	0.79
Self-Confidence	0.77	0.84
Self-Fear Management	0.63	0.71

**Table 2:** Basic description of the sample.

N = 117		n (s.d)
Gender	Female	95 (81.2)
	Male	22 (18.8)
Average age (1st/4th year)		20.31/23.3 (4.2)
Average EBBCAM (1st/4th year)	A&C	42.83/48.90
	PMC	31.32/33.82
	SC	32.13/42.59
	MSF	43.93/48.86
	**1st grade**	**4th grade**
		**n (s.d)**
Average interest in death	6.93	7.19
High interest	90 (76.9)	94 (80.3)
Low interest	26 (22.4)	23 (19.7)

A&C: Accompaniment and Communication; PMC: Post-Mortem Care; SC: Self-Confidence; MSF: Management of Self-Fear. s.d. standard deviation.

**Table 3:** Gender comparison.

	1st Year		4th Year	
	Gender (f/m)	*p*	Gender (f/m)	*p*
Accompaniment and Communication	42.21/45.53	0.190	49.26/47.34	0.451
Post-Mortem Care	30.45/35.11	0.136	34.03/32.95	0.726
Self-Confidence	32.07/32.42	0.915	43.26/39.70	0.168
Self-Fear Management	42.91/48.33	0.065	49.51/46.06	0.184

f: female; m: male.

**Table 4:** Comparison per degree.

	Mean 1st/4th Degree	*p*
Accompaniment and Communication	42.30/48.90	0.000
Post-Mortem Care	31.32/33.82	0.077
Self-Confidence	32.14/42.59	0.000
Self-Fear Management	43.93/48.86	0.001

**Table 5:** Adjustment of the variables analysed to the predictor model.

Coefficient	Non-Standardized		Standardized		
Model	B	Standard Error	Beta	T	Sig.
Constant	51.017	9.375		5.442	0.000
Age	0.013	0.312	0.004	0.043	0.966
Interest	−0.109	0.764	−0.013	−0.143	0.886
Gender	−6.336	3.376	−0.170	−1.877	0.063
Competence	0.449	0.107	0.399	4.197	0.000

B: coefficient; sig: significance; T: Student´s t distribution.

**Table 6:** Influence of first-year scores on fourth-year scores.

	R	R^2^	Adjusted R^2^	Model Sig.
Total competence	0.374	0.140	0.132	0.000 *
Accompaniment and Communication	0.351	0.123	0.115	0.000 *
Post-Mortem Care	0.323	0.104	0.097	0.000 *
Self-Confidence	0.213	0.045	0.037	0.021 *
Self-Fear Management	0.050	0.003	−0.006	0.592

* ANOVA < 0.001; R, R^2^: correlation coefficient, square; Sig: significance

**Table 7:** Correlations between EBBCAM and participants’ age and interest in death.

	First Grade				Fourth Grade			
	A&C	PMC	SC	MSF	A&C	CPM	SC	MSF
**Age**								
*r*	0.012	0.101	−0.141	0.131	−0.038	0.038	−0.030	−0.017
*p*	0.901	0.285	0.132	0.164	0.690	0.683	0.751	0.857
**Interest**								
*r*	0.250	0.117	0.281	0.324	0.077	0.130	0.233	0.115
*p*	0.007	0.213	0.002	0.000	0.410	0.161	0.011	0.216

A&C: Accompaniment and Communication; PMC: Post-Mortem Care; SC: Self-Confidence; MSF: Management of Self-Fear.

## Data Availability

The data presented in this study are not publicly available.
